# Prevalence of oral mucosal lesions in psoriatic patients: A controlled study

**DOI:** 10.4317/jced.50905

**Published:** 2012-12-01

**Authors:** Azmi MG. Darwazeh, Mustafa M. Al-Aboosi, Ahmad A. Bedair

**Affiliations:** 1 BDS, MSc, PhD, FFDCRSI. Professor, Department of Oral Medicine and Surgery, Faculty of Dentistry; 2 PhD. Associate Professor, Department of Internal Medicine and Dermatology, Faculty of Medicine, Jordan University of Science & Technology, Irbid, Jordan; 3 BDS, MSc. Zarqa Governate Health Directorate, Ministry of Health, Jordan

## Abstract

Objectives: This study aimed to investigate and compare the prevalence of oral mucosal lesions in a group of psoriatic patients and healthy subjects, and its correlation to multiple clinical parameters. Study design: 100 psoriatic patients and 100 closely matched controls underwent clinical oral examination. Oral lesions were diagnosed according to the criteria proposed by the World Health Organization (WHO). The patients filled the Hospital Anxiety and Depression (HAD) questionnaire and the Dermatology Life Quality Index (DLQI). The severity of psoriasis was assessed by the Psoriasis Area and Severity Index (PASI). Categorical variables were evaluated using Chi-square test or Fisher’s exact test with overall significance set at p< 0.05. Results: Oral mucosal lesions were diagnosed in 43 (43%) psoriatic patients and 17 (17%) control subjects (p=0.000). Comparing psoriatic patients to control subjects the prevalence of fissured tongue (FT) was 35% vs. 13% (p=0.000); geographic tongue (GT) 17% vs. 9% (p=0.09); combination of FT and GT 5% vs. 5% (p=1.00); oral candidosis 3% vs. 0% (p=0.81); leukoedema 1% vs. 3% (p=0.62); physiologic melanin pigmentations 4% vs. 1% (p=0.37) respectively. The clinical type of psoriasis, duration of the disease, method of disease management (medicated vs. non-medicated for psoriasis), smoking habit, psychological status or the disease severity did not influence the prevalence of FT and GT. Psoriatic patients who experienced “very large” to “extremely large” adverse effect of psoriasis on their quality of life have significantly higher prevalence of GT (p=0.04). Conclusions: FT is significantly more common in psoriatic patients compared to controls; hence studies investigating the nature of this relationship are warranted. Oral health care providers should be aware of the predisposition of psoriatic patients to oral candidosis.

** Key words:**Oral lesions, fissured tongue, geographic tongue, leukoedema, oral Candida, candidosis, psoriasis.

## Introduction

Psoriasis is a common multisystem inflammatory chronic disease with predominantly skin and joint manifestations. The estimated prevalence of psoriasis ranges between 0.5% and 4.6% with geographic and ethnic variations (1). Psoriasis is classified clinically into several types such as plaque type (also known as psoriasis vulgaris), inverse, erythrodermic, pustular, guttate, psoriatic onychodystrophy and psoriatic arthritis. The plaque type is the most common form of the disease affecting up to 90% of the cases (1).

The dermatological and joint manifestations of the disease are well documented, but whether psoriasis has oral manifestations (2,3), or not (4,5) is still a subject of controversy. Various oral lesions, such as geographic tongue (GT) (2,6-8), fissured tongue (FT) (2,3,9), and to less extent leukoedema (LD) and physiologic melanin pigmentation (3), have been described with higher prevalence in psoriatic patients compared to control subjects. Oral mucosal psoriasis, as proved by histopathological testing, has been recently reported in patients without cutaneous psoriatic lesions (10).

Reviewing the literature reveals lack of consensus regarding the association of oral lesions with a certain clinical type of skin psoriasis (2,3,9,11). One study suggested a positive correlation between the prevalence of oral lesions and the severity of the psoriasis as assessed by the Psoriasis Area and Severity Index (PASI) (9). The psoriasis is known to have a profound impact on the quality of the patient’s life (12), but the relationship of oral lesions to the Dermatology Life Quality Index (DLQI) has not been evaluated in psoriatic patients before. Therefore, the aims of this study were to assess the prevalence of oral mucosal lesions in psoriatic patients in comparison with a control group closely matched for age and gender, and to explore any association with the type of psoriasis, duration of psoriasis, severity of the disease, disease onset, type of psoriasis treatment, and tobacco smoking habit. The study also aimed to evaluate the psychological status of psoriatic patients (presence of anxiety and depression), and patient’s life quality as a sequence to psoriasis and their relationship to the oral lesions.

## Material and Methods

The subjects of the study group were adult patients diagnosed with psoriasis by their dermatologist, recruited consecutively from patients attending the Dermatology Clinics at King Abdullah University Hospital (KAUH) and Princess Basmah Hospital (PBH) in the city of Irbid located in North of Jordan. The exclusion criteria was history of antibiotic intake over the past two months, regular use of antiseptic mouth wash, wearing removable dental prosthesis, pregnancy, anemia, or diabetes mellitus. Subjects with a current or history of any dermatological disease (other than psoriasis for the study group) were also not included. The control subjects closely matched for age and gender were selected from the hospital staff and the companions of the patients at KAUH who were biologically not related to the study group subjects. The control subjects fulfilled the same selection criteria for the study group except the history of psoriasis.

A subject was classified as “smoker” when he / she reported smoking at least one cigarette daily. The “non-smoker” was defined as either never had smoked, or who had quit smoking for at least one year prior to the study (13). The Hospital Anxiety and Depression (HAD) questionnaire (14), and the Dermatology Life Quality Index (DLQI) (15) was filled by psoriatic patients.

Clinical oral examination was carried out by a one of the authors (AAB) while the subject seated on a chair using artificial light and sterile mirrors, and the diagnosis of oral mucosal lesions was made on a clinical basis according to the method and criteria proposed by WHO (16). The swab and smear tests were performed on lesions suggestive for oral candidosis. The swabs were streaked on Sabouraud’s dextrose agar plates (Oxoid Ltd, Basingstoke, England) and incubated aerobically at 37º C for 48 hours. Candida colonies were identified based on the colony color, texture and morphology, and determination of the purity of the culture was then performed microscopically by the wet mount technique at X40 objective. Candida species were identified using the germ-tube test (17) and the VITEK 2® Identification System using the new colorimetric yeast cards (18) (BioMerieux Inc. Durham NC, USA). Smears were Gram stained and examined under light microscope at X40 objectives for the presence of fungal hyphae or blastospores. Candida isolation and identification was performed in the Microbiology Laboratory in KAUH. Oral candisosis was finally diagnosed when the subject exhibited the clinical signs and symptoms of oral candidosis in addition to the positive isolation of Candida species from the swab culture samples, and Candida blastospores or hyphae were detected in the stained smear.

The severity of psoriasis was assessed by the Psoriasis Area and Severity Index (PASI) score which was calculated for the psoriatic patient by the treating dermatologist. According to the PASI score the severity of psoriasis was categorized as “mild” when the total score calculated was < 10, “moderate” when the total score was within the range of 10-20 and “severe” when the total score was > 20 (19). The DLQI (15) categorizes the effect of psoriasis on the quality of the psoriatic patient life of into:

1) No effect: score = 0-1

2) Small effect: score = 2-5

3) Moderate effect: score = 6-10

4) Very large effect: score = 11-20

5) Extremely large effect: score = 21-30

All patients were interviewed concerning their relevant medical and social history.

This study protocol was approved by the Institutional Review Board (IRB) at KAUH in compliance with the Helsinki Declaration, and all subjects signed the consent form which was also approved by the IRB.

Data were analyzed using the Statistical Package for the Social Sciences (SPSS) version 17.0. Categorical variables were evaluated using Chi-square test or Fisher’s exact test with overall significance set at p < 0.05.

## Results

Each of the study and control groups consisted of one hundred subjects with a mean age of 32.9 ± 14.8 years. Each group consisted of 54 males (54%) and 46 females (46%). The mean duration of psoriasis was 10 ± 8.5 years ranging between 1.5 months and 43 years.

Oral mucosal lesions were diagnosed in 43 (43%) psoriatic patients and 17 (17%) control subjects (p=0.000). Comparing psoriatic patients to control subjects the prevalence of fissured tongue (FT) was 35% vs. 13% (p=0.000); geographic tongue (GT) 17% vs. 9% (p=0.09); combination of FT and GT 5% vs. 5% (p=1.00); oral candidosis 3% vs. 0% (p=0.81); leukoedema 1% vs. 3% (p=0.62); physiologic melanin pigmentations 4% vs. 1% (p=0.37) respectively. Due to relatively low prevalence of some oral lesions, further statistical analysis and associations were only to the results of FT and GT.

The three psoriatic patients who showed clinical and microbiologic evidence of oral candidosis were two cases of erythematous candidosis and one case of median rhomboid glossitis, and all caused by Candida albicans. These three patients were under topical betamethasone (Daivobet®) therapy, of the plaque type psoriasis, and non-smokers, and were subsequently referred for treatment.

Due to the small number of patients presented with types of psoriasis other than the plaque type (≤ 5 patients), the patients were divided into two main subgroups; the plaque type and non-plaque type psoriasis. The results showed that the clinical type of psoriasis, the duration of the disease or the method of disease management (medicated vs. non-medicated for psoriasis), or the smoking habit did not influence the prevalence of FT and GT ( Table 1).

**Table 1:**
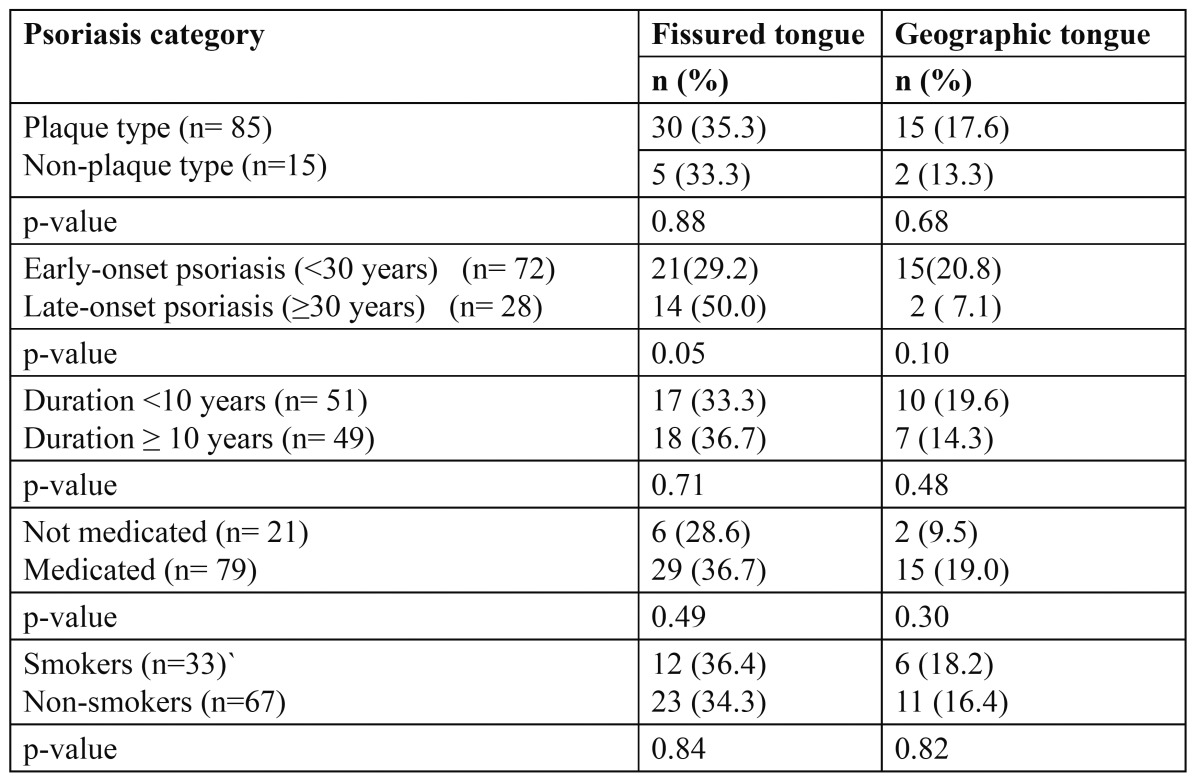
The relationship of fissured tongue and geographic tongue to the different clinical parameters in psoriatic patients.

Only 87 psoriatic patients and 88 control subjects completed the HAD scale form. The psoriatic patients with categorized with significant level of depression and anxiety had relatively higher prevalence of FT and GT but this have not reached significance ( Table 2).

**Table 2:**
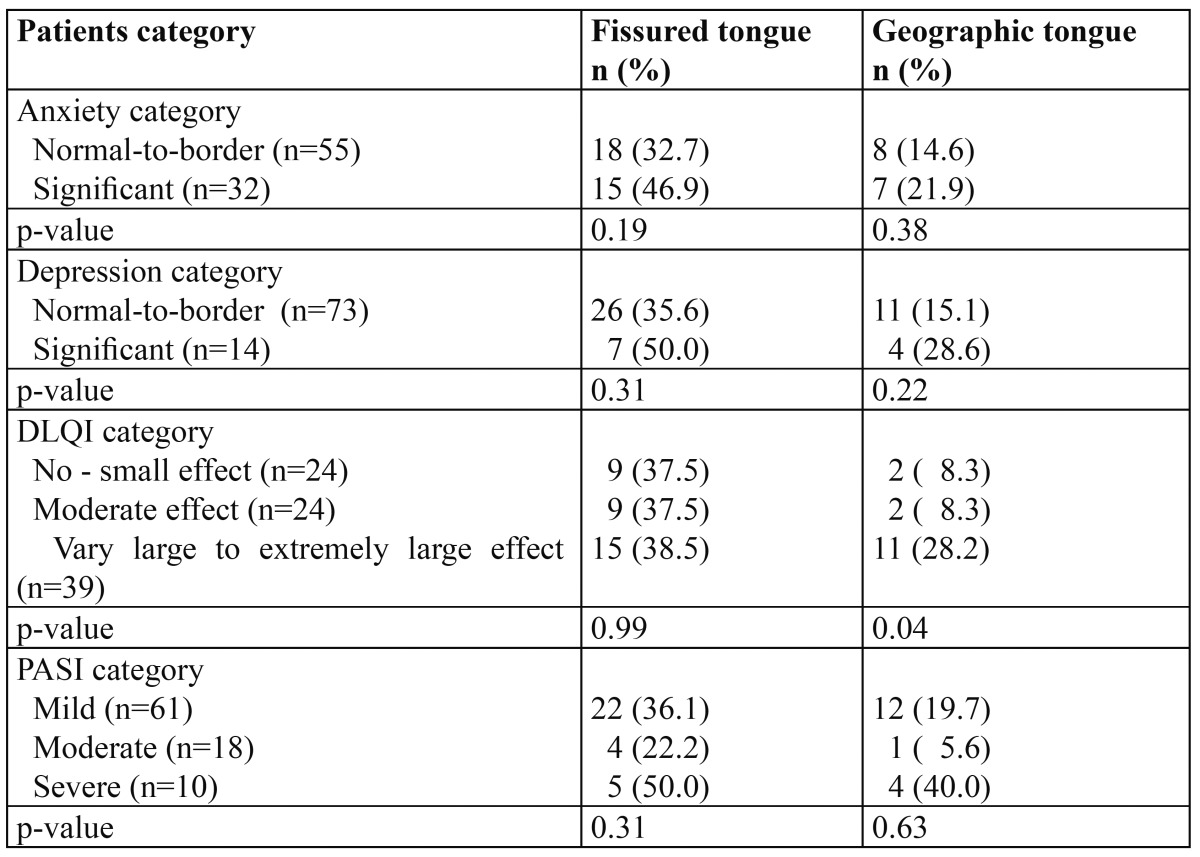
The relationship of fissured tongue and geographic tongue to the various categories of psoriatic patients.

The prevalence of FT and GT was also relatively higher among patients with severe psoriasis as assessed with PASI scores, nevertheless the difference was not significantly different that the prevalence in less severe cases ( Table 2). Psoriatic patients who experienced very large to extremely large adverse effect of psoriasis on their quality of life have significantly higher prevalence of GT (p= 0.04;  Table 2).

## Discussion

Despite psoriasis is a common skin disorder, reviewing the literature reveals that reports of it involving the oral mucosa are relatively rare, with less than 100 publications (20). In addition, weather these oral mucosal lesions are representing true oral manifestations of the disease (10), or not (4,20) is still controversial. This is possibly because mucosal biopsy is seldom done for these oral lesions, and the correlation between the oral lesions and the clinical course of skin psoriasis has not been established. Our closely-matched controlled study has lent support to the theory that psoriatic patients in general are presented with more oral lesions than non-psoriatic subjects (21).

According to the results of our study on this group of Jordanian psoriatic patients, FT was the most common oral finding, and significantly more frequent in psoriatic patients than controls, which confirms the results of other studies in other populations (2,3,21,22). Our findings that 35% prevalence of FT in psoriatic patients falls within the range of 6% to 47.5% reported in different studies (2,3,6,9,22,23) and in accordance to Costa et al. (2) and Germi et al. (21) who reported FT in 34.3% and 35.1% of their psoriatic patients respectively. In addition, the prevalence of FT among our control subjects (13%) was very close to that previously reported among Jordanians (11.5%) (24), which gives validity to the results.

Considering the possibility of the presence of a shared genetic basis for psoriasis and FT is one of the possible explanations for the frequent occurrence of FT in psoriatic patients (2), and encouraged the researchers to hypothesize that FT may be an oral manifestation of psoriasis (2). Such conclusion should be considered with caution since a recent study could not demonstrate a common genetic factor in psoriasis and FT (25), and the histopathological or clinical parallelism with the cutaneous psoriasis is still unproven. The precise relationship of FT to psoriasis is still unclear. The finding of marginally significant high prevalence of FT in patients with late onset psoriasis compared to early onset disease (50% and 29.2% respectively; p=0.05) may be partly explained by the reported successively increasing prevalence of FT with advancing age (24).

The prevalence of GT in our psoriatic patients (17%) is slightly higher than the prevalence range reported in other studies (1.3%-14.0%) (3,6,8,9,22,23). This may be due to differences in age, gender and type of psoriasis distribution in the population studied. However, the precise prevalence of GT may be difficult to determine since this study, and the other studies, were cross-sectional, and cases of GT are known to be subjected to flare ups and remissions with time. Therefore, future longitudinal, rather than cross-sectional, studies are encouraged if the relationship between GT and psoriasis is to be elucidated.

Consistent with our results, Shulman and Carpenter (4) and Miloğlu et al. (5) could not find a significant difference in the prevalence of GT between psoriatic patients and control subjects. On the contrary, multiple surveys reported a significant higher prevalence of GT in psoriatic patients (2,7,9). One histopathological study biopsied GT lesions in psoriatic patients and control subjects and described typical features of psoriasis in all cases from psoriatic patients but in 80% of the control subjects, hence concluded that GT is an expression of oral psoriasis (7).

There was much debate concerning the relationship between the psoriasis clinical type and oral lesions. Costa et al. (2) could not find an association between the type of psoriasis and the presence of oral lesions which is consistent with our study. Some studies reported higher prevalence of FT in generalized pustular psoriasis (9,11), psoriasis erythroderma (9) and plaque type psoriasis (3). However, the analysis of the prevalence of oral lesions by psoriasis clinical type was limited in the current study since the patients were divided as plaque type and non-plaque type due to the small number of patients presented with types of psoriasis other than the plaque type.

Tobacco smoking is associated with numerous dermatologic conditions including psoriasis (26). The prevalence of FT and GT in our psoriatic patients was almost equal in non-smokers and smokers which was also the finding of other studies (3), while others found that GT had a low prevalence in smokers (22).

Within this limited number of psoriatic patients, there is a tendency for the prevalence of FT and GT to correlate positively, though not significantly, with the patient’s anxiety and depression category. GT is significantly higher in patients whom psoriasis had severe impact on their life. In general, it is widely accepted that GT lesions exacerbate after stressful events and this implies that this lesion is related to emotional stress with a threshold of susceptibility to anxiety (27). Nevertheless, we could not detect a significant positive correlation between the severity of the psoriasis and the prevalence of any of the oral lesions on contrary to Daneshpazhooh et al. (9) who reported increased frequency of GT with increased severity of psoriasis only in patients with plaque type psoriasis.

The literature on the oral candidosis in patients with psoriasis is limited. Henseler and Tausch (28) reviewed the medical records of 44695 dermatological patients and concluded that psoriatic patients had 1.3-1.6 relative risk of developing mucocutaneous candidosis compared to healthy persons. Skinner et al. (29) reported 14 psoriatic patients in whom the disease was associated with oro-pharyngeal Candida. Angular cheilitis was reported in 11% (23) and erythematous candidosis and pseudomembranous candidosis (3) in 7.5% and 1.2% of the psoriatic patients respectively. Our finding of oral candidosis in 3% of psoriatic patients but in none of the healthy control subjects supports the view that psoriatic patients may be predisposed for oral candidosis (3). It is noteworthy that all the three cases were on topical betamethasone (Daivobet®) therapy for their skin disease. Nevertheless, this finding needs to be confirmed and the predisposing factor(s) to be identified.

In conclusion, there is a consensus that some oral mucosal lesion such as FT and GT are strongly associated with psoriasis. Although these lesions are non-pathognomonic to psoriasis, their precise relationship needs to be clarified, and weather they constitute the oral counterpart of the cutaneous lesions await further studies. Oral health care provider should aware of the liability of psoriatic patients to oral candidal infection.
